# Supplementary figures and images for: Serial ‘deep-sampling’ PCR of fragmented DNA reveals the wide range of Trypanosoma cruzi burden among chronically infected human, macaque, and canine hosts, and allows accurate monitoring of parasite load following treatment
Source: eLife. 2025 Apr 15;14:RP104547. doi: 10.7554/eLife.104547 (PMC11999692; doi:10.7554/eLife.104547)

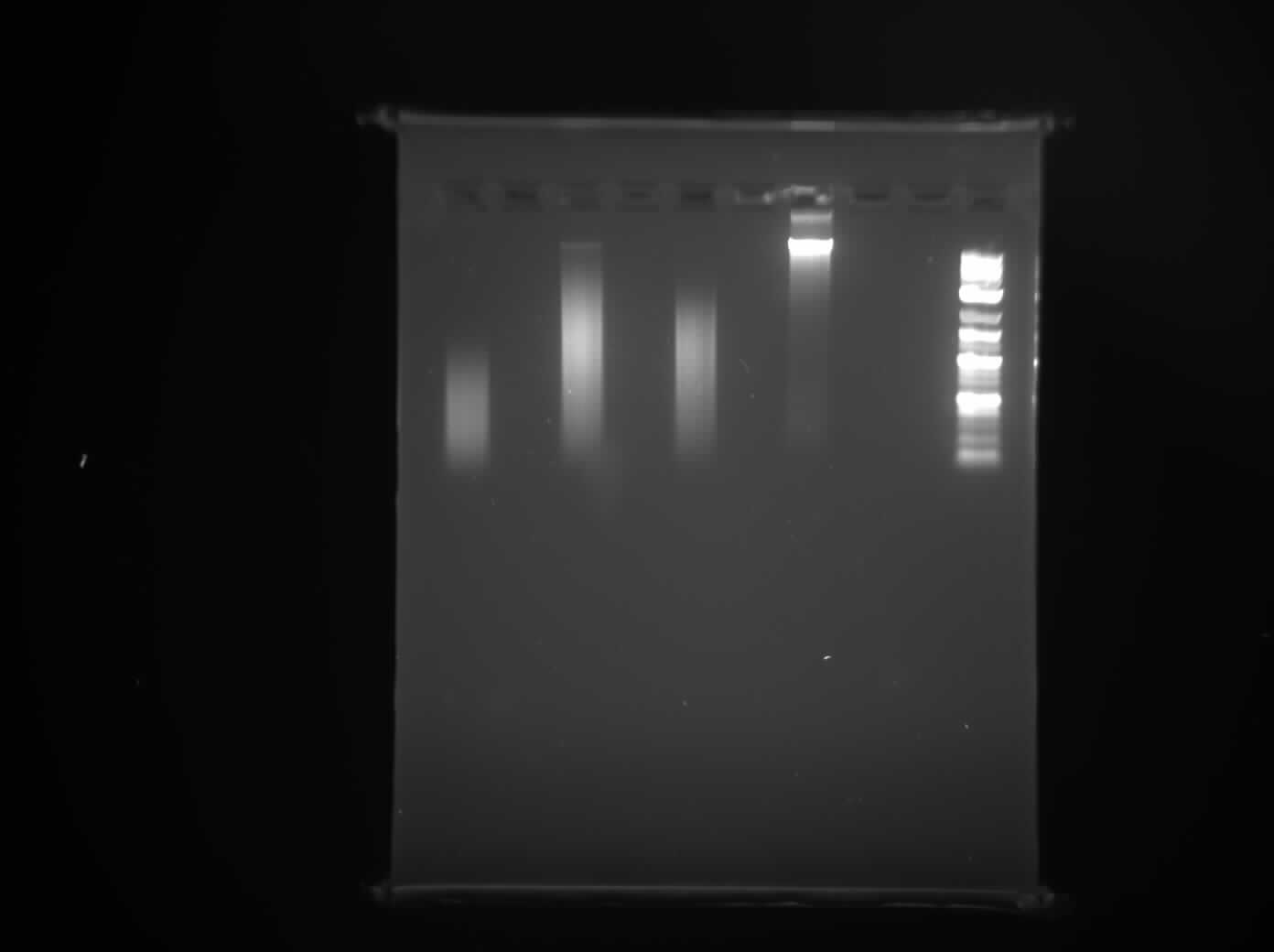

Supplement: Figure 4—figure supplement 1—source data 2. [file elife-104547-fig4-figsupp1-data2.zip › 6.12.23c.jpg]

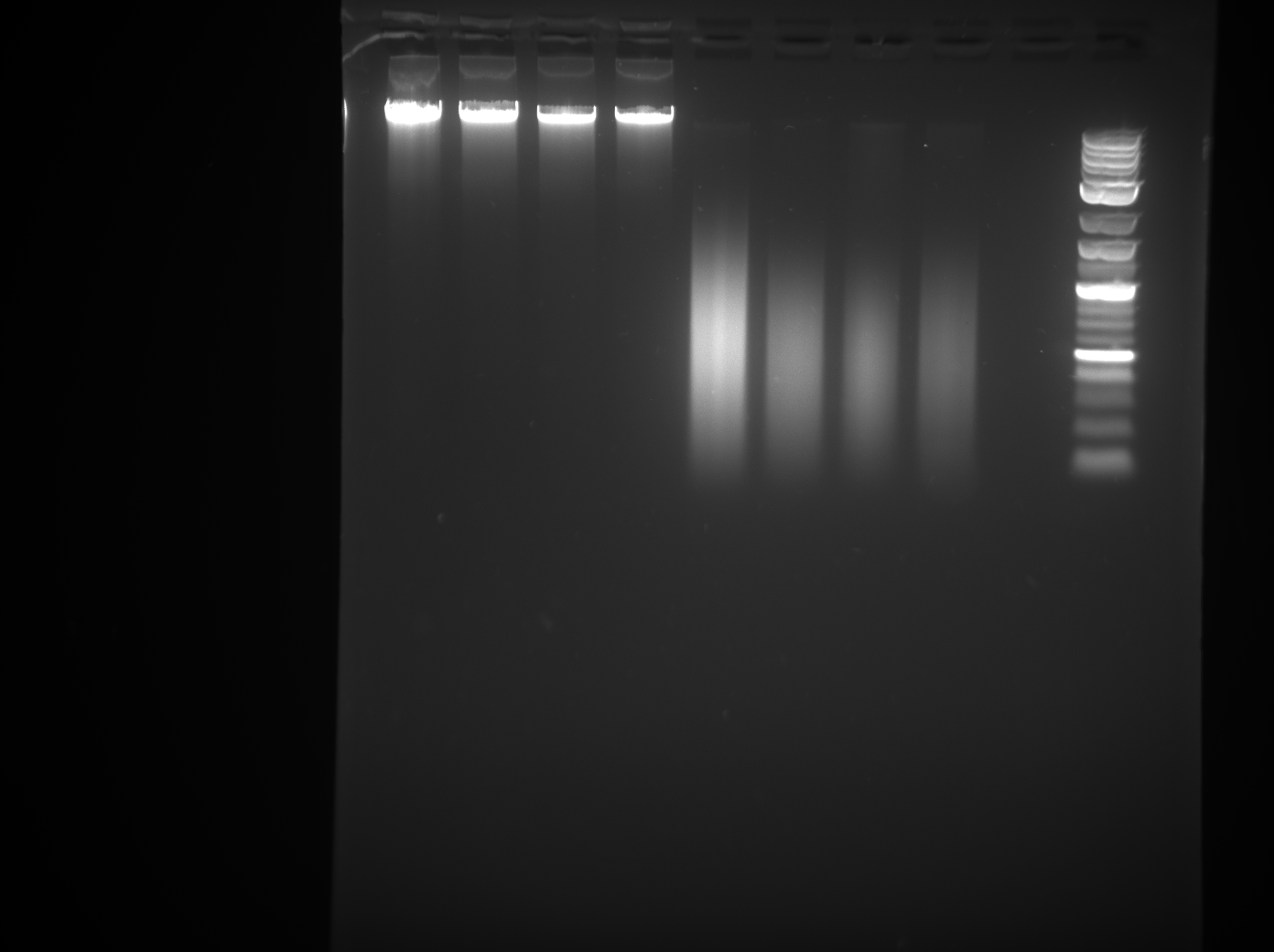

Supplement: Figure 4—figure supplement 1—source data 2. [file elife-104547-fig4-figsupp1-data2.zip › 8.10.23FRAGBLOOD.tif]

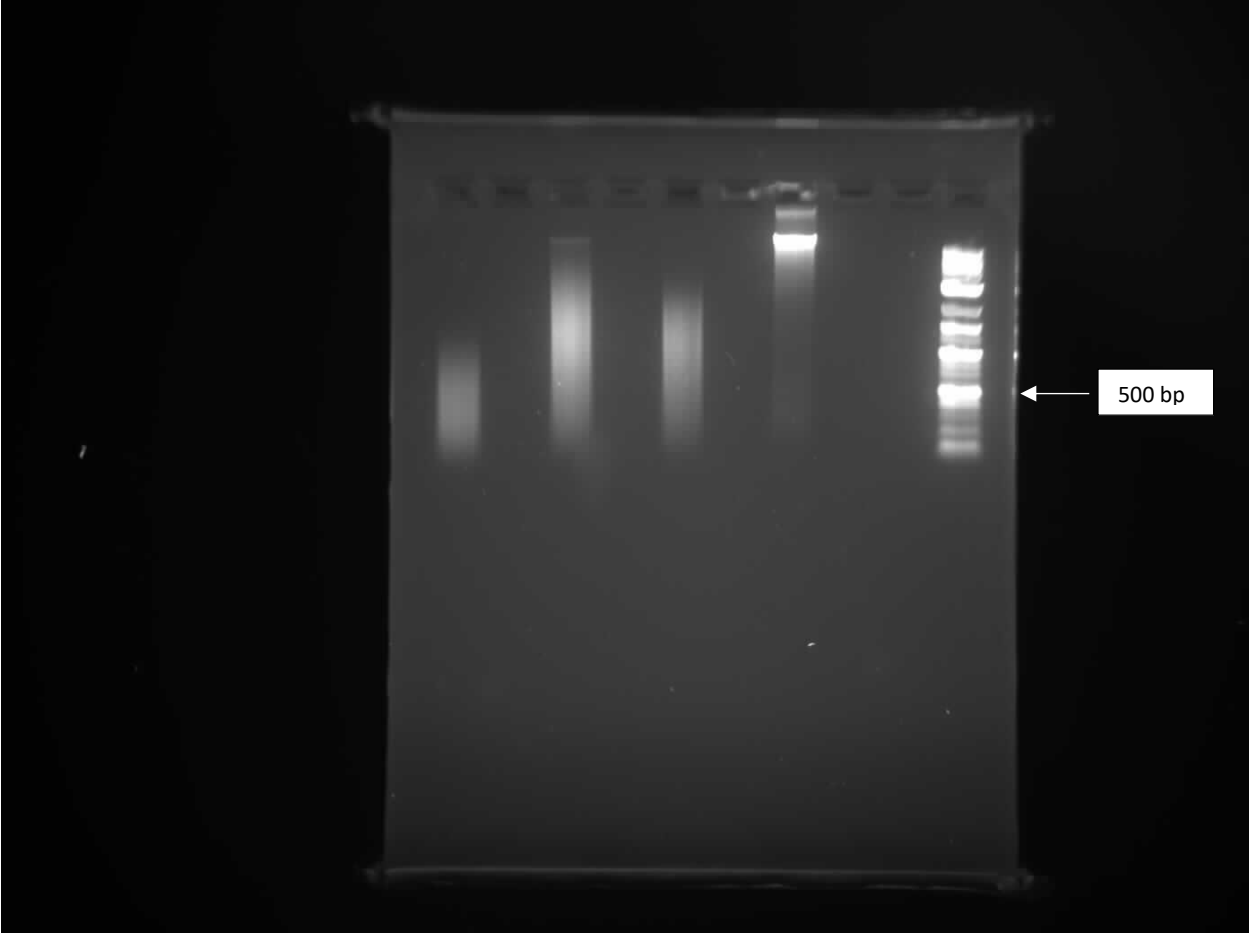

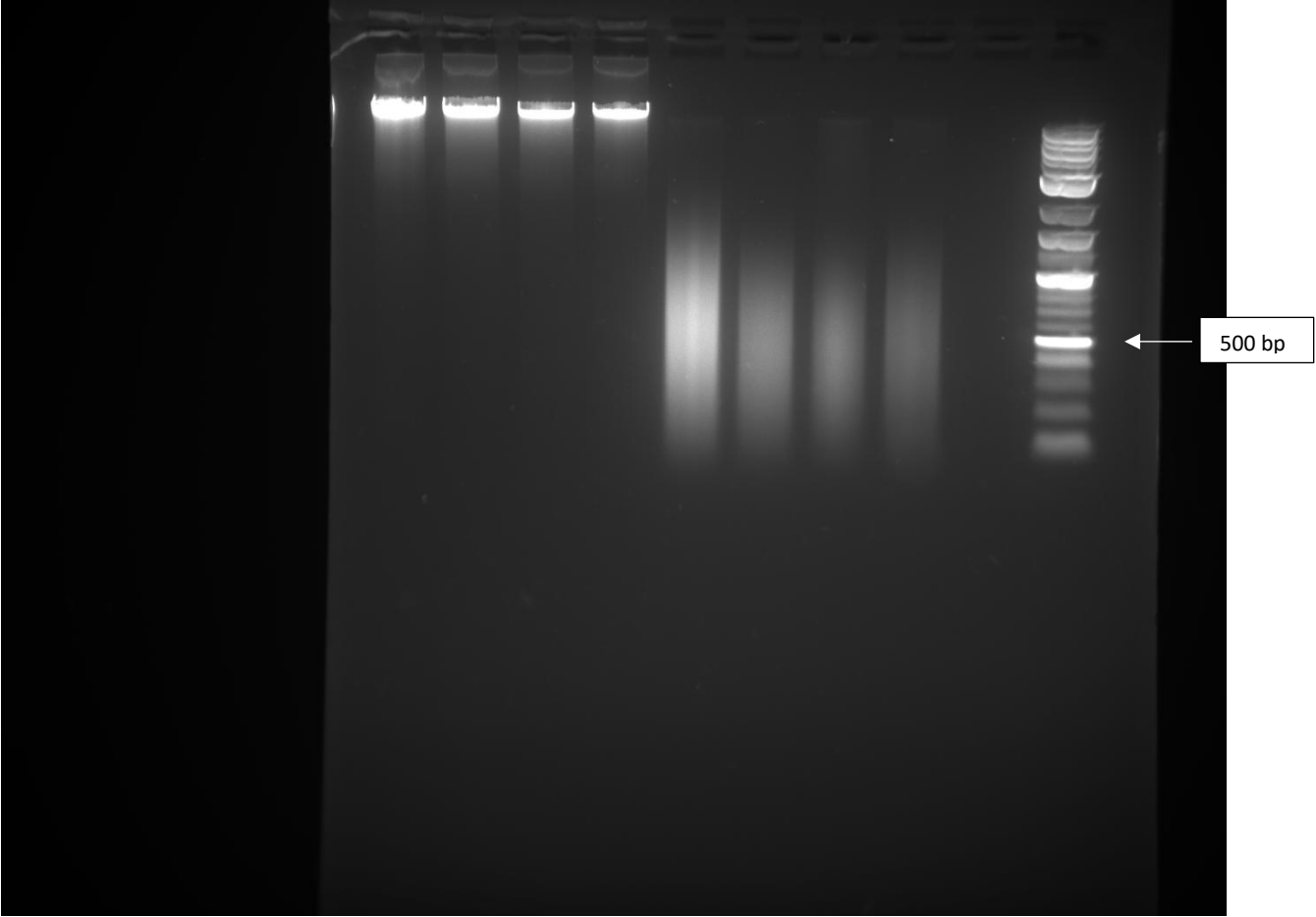

Supplement: Figure 4—figure supplement 1—source data 3. [file elife-104547-fig4-figsupp1-data3.pdf]
