# Supplementary material for: Serial ‘deep-sampling’ PCR of fragmented DNA reveals the wide range of Trypanosoma cruzi burden among chronically infected human, macaque, and canine hosts, and allows accurate monitoring of parasite load following treatment
Source: eLife. 2025 Apr 15;14:RP104547. doi: 10.7554/eLife.104547 (PMC11999692; doi:10.7554/eLife.104547)
Supplement: Supplementary file 1. — N1 and N2 are seronegative controls and macaques T1–T5 were previously infected but cured of T. cruzi infection using benzoxaborole AN15368 (Padilla et al., 2022). [file elife-104547-supp1.docx]

Supplementary File 1. Historical and year one PCR and hemoculture summary for macaques

| **ID** | **Sex** | **Birth Year** | **Year Infected/ Sero-converted** | **Age (yr) at infection** | **First confirmed conventional PCR pos.** | **Total number of PCR reactions (up to 12 months)** | **0verall % deep-sampling PCR pos** | **Ave positive Cq value** | **Overall % pos Hemoculture** |
| --- | --- | --- | --- | --- | --- | --- | --- | --- | --- |
| **P1** | M | 1999 | 2013 | 14 | 2016 | 60 | 70.00 | 31.92 | 54.3 |
| **P2** | M | 2000 | 2015 | 15 | 2016 | 3366 | 0.56 | 33.42 | 3.3 |
| **P3** | F | 2001 | 2013 | 12 | 2016 | 1,869 | 2.09 | 32.84 | 15.0 |
| **P4** | F | 2004 | 2014 | 10 | 2018 | 316 | 3.16 | * | * |
| **P5** | M | 2004 | 2015 | 11 | 2016 | 60 | 81.67 | 30.39 | 63.3 |
| **P6** | M | 2005 | 2012 | 7 | 2018 | 1784 | 5.60 | 33.71 | 1.7 |
| **P7** | F | 2005 | 2015 | 10 | 2018 | 3062 | 0.29 | 35.90 | 0 |
| **P8** | F | 2009 | 2014 | 5 | 2016 | 1980 | 1.31 | 34.07 | 5.7 |
| **P9** | M | 2009 | 2017 | 8 | Mult.neg. 2018 | 3256 | 0.21 | 33.44 | 1.7 |
| **P10** | F | 2011 | 2017 | 6 | 2018 | 712 | 4.77 | 32.66 | 15.0 |
| **P11** | M | 2012 | 2021 | 9 | no PCR data | 2888 | 1.76 | 35.03 | 15.0 |
| **P12** | M | 2013 | 2018 | 5 | no PCR data | 1592 | 0.25 | 31.20 | 0 |
| **P13** | M | 2013 | 2018 | 5 | no PCR data | 438 | 24.20 | 32.72 | 6.7 |
| **P14** | F | 2013 | 2021 | 8 | no PCR data | 70 | 52.86 | 29.70 | 73.3 |
| **P15** | F | 2014 | 2020 | 6 | no PCR data | 70 | 70.00 | 28.47 | 57.1 |
| **P16** | F | 2015 | 2021 | 6 | no PCR data | 70 | 81.42 | 25.59 | 48.6 |
| **P17** | M | 2015 | 2019 | 4 | no PCR data | 80 | 68.75 | 25.39 | 62.9 |
| **P18** | F | 2015 | 2019 | 4 | no PCR data | 60 | 56.70 | 32.11 | 46.7 |
| **P19** | F | 2015 | 2021 | 6 | no PCR data | 60 | 48.33 | 32.16 | 20.0 |
| **P20** | F | 2015 | 2021 | 6 | no PCR data | 70 | 40.00 | 32.64 | 43.3 |
| **P21** | F | 2015 | 2019 | 4 | no PCR data | 70 | 24.28 | 32.54 | 10.0 |
|  |  |  |  |  |  |  |  |  |  |
| **T1** | F | 2001 | 2011 | 10 | 2016 | 2310 | 0 |  | 0 |
| **T2** | F | 2005 | 2014 | 9 | 2016 | 1925 | 0 |  | 0 |
| **T3** | F | 2006 | 2015 | 9 | 2016 | 1925 | 0 |  | 0 |
| **T4** | F | 2006 | 2015 | 9 | 2016 | 1925 | 0 |  | 0 |
| **T5** | F | 2012 | 2016 | 4 | 2016 | 2310 | 0 |  | 0 |
|  |  |  |  |  |  |  |  |  |  |
| **N1** | F | 2009 | Never | NA | no PCR data | 2346 | 0.04 |  | 0 |
| **N2** | F | 2013 | Never | NA | no PCR data | 2343 | 0 |  | 0 |

*Died during study - chronic colitis, possible early colon cancer/precancerous lesion
